# The first assessment of grain yield and associated traits in durum wheat across a decade in Nepal

**DOI:** 10.3389/fpls.2024.1456062

**Published:** 2024-10-14

**Authors:** Dhruba Bahadur Thapa, Mahesh Subedi, Manoj Sapkota, Suman Bohara, Keshab Raj Pokhrel, Laxman Aryal, Basistha Acharya, Santosh Tripathi, Chhotelal Chaudhary, Bramanti Mahato, Krishna Timsina, Velu Govindan, Arun Kumar Joshi

**Affiliations:** ^1^ Nepal Agricultural Research Council (NARC), Kathmandu, Nepal; ^2^ Breeding Insight, Cornell University, Ithaca, NY, United States; ^3^ International Maize and Wheat Improvement Center (CIMMYT) and Borlaug Institute for South Asia (BISA), New Delhi, India

**Keywords:** durum wheat, yield, genetic variation, cluster analysis, breeding

## Abstract

Rapid urbanization and evolving dietary preferences have heightened the demand for durum wheat and its derivatives in developing nations like Nepal. This study represents the first comprehensive exploration and evaluation of durum wheat genotypes in Nepal, addressing the escalating need for high-yielding varieties. The primary objective was to identify stable and prolific durum wheat lines for release, enhancing Nepal’s durum wheat breeding program. Utilizing genotypes from CIMMYT’s disease screening and yield nurseries from 2011/12 to 2020/21, a total of 132 genotypes, including international checks, underwent evaluation over ten years under the Alpha Lattice design. Results revealed significant variation among genotypes for grain yield and other traits, identifying high-yielding and stable lines suitable for Nepal. Heritability analysis highlighted moderate heritability for grain number per spike, thousand-grain weight, and grain yield. Cluster analysis identified distinct clusters with high grain yield and desirable agronomic traits. Disease incidence facilitated the selection of resistant lines, with DWK38 emerging as the highest grain yielder (4416.04 kg/ha). Overall, durum wheat lines from CIMMYT exhibited robust performance in Nepal, enabling the identification of superior lines with potential benefits for farmers and consumers. The study’s implications include developing and releasing superior durum lines in Nepal, providing farmers with profitable alternatives amidst evolving food habits. In conclusion, the findings from this study provide a valuable foundation for future durum wheat breeding efforts in Nepal, guiding the selection of genotypes that are well-suited to the diverse environmental challenges of the region.

## 1 Introduction

Durum wheat evolved from domesticated emmer wheat (*T. turgidum* spp. *dicoccum* (Schrank ex Schübl.) Thell. originated from wild emmer wheat (*T. turgidum* spp. *dicoccoides* Körn. ex Asch. & Graebn.) in the Fertile Crescent approximately 10,000 years ago (Özkan et al., 2002; Dubcovsky and Dvorak, 2007). Over the years, durum wheat continued to evolve from the domestication of wild to emmer wheat, followed by durum wheat landraces, and modern breeding achieved further improvements (Maccaferri et al., 2019). Durum wheat is grown globally on 13.5 million hectares, which amounts to about 6.2% of the wheat area, with a global production of 33.8 million tons in 2020/21 (Dahl, 2022; IGC, 2022). However, durum wheat is no longer just a staple crop for food security but has become a major cash crop. It provides the raw material for semolina, pasta, couscous, bulgur, and several other dishes of Mediterranean tradition (Oliveira et al., 2012). The pasta, burghul, and couscous industries currently purchase durum grain at prices 10 to 20% higher than bread wheat, indicating significant scope for expanding the durum wheat area. However, this will depend on the market’s ability to purchase durum wheat at a higher price to stimulate farmer adoption (Tidiane Sall et al., 2019).

The endosperm of durum wheat grain has a glassy texture and is hard to crush. The grains are large and elongated with high protein content and amber-colored vitreous endosperm. On milling, grain breaks to form a uniform large granular product called semolina, which is highly valued for preparing premium pasta products, diverse food products like bread, bulgur, biscuits, noodles, and high-quality pasta products (Abecassis et al., 2012). Pasta and couscous are the most common food products made from durum wheat (Hammami and Sissons, 2020). Historically, it is well adapted to low and variable rainfall environments with frequent terminal heat stress, such as the Mediterranean Basin (Algeria, Turkey, Italy, Morocco, Syria, Tunisia, France, Spain, and Greece), which account for approximately 50% of world acreage and production. Besides these countries, Canada, Mexico, USA, Russia, Kazakhstan, Azerbaijan, and India are large durum wheat producers. Canada, Mexico, and the USA are large exporters of durum wheat, whereas Nigeria, Egypt, and China are major importers of durum wheat (World Bank, 2021).

Based on annual data of the Department of Customs, Government of Nepal, Nepal has imported 1340 tons of Durum wheat grain worth more than $400,000 within the period of mid July 2021 to mid-July 2022. Similarly, Nepal imported 2462 tons of pasta worth $300,000 and more than 2 tons of couscous in the fiscal year 2021/21 (Ministry of Finance, 2022). Importing durum wheat grains and products like pasta in the country indicates a greater scope of durum wheat production and product diversification. Plain areas of western Nepal have a favorable environment for durum cultivation (ABD, 2014). Studies have shown that durum wheat can produce as high as 6.7 tonnes of grain yield per hectare of land in Banke district, Nepal (DOAR, 2021). China, the neighboring country and one of the greatest importers of durum wheat (World Bank, 2021), has opened the internal market scope for Nepalese durum wheat. In European countries, durum wheat price is 5-25% higher than bread wheat (Ranieri et al., 2012). Similarly, durum wheat pasta and macaroni in the Nepalese market are fetching higher prices than other pastas. With changes in the food habits of urban and semi-urban people and the nutritional value of durum wheat products, the demand for durum products is increasing in the country.

Wheat production on farms will have to increase significantly to meet the future demand and coup of climate change, which poses a risk to even current production rates (Beres et al., 2020). There are pronounced stresses on the production of quality durum wheat, namely abiotic (drought, frost, sprouting) and biotic (disease, insect pests, weed). Apart from yield and pasta making quality, the breeding of durum wheat for developing micronutrient (Fe and Zn) enriched varieties has occurred globally. A very positive association between grain protein concentration, Fe, and Zn has been confirmed (Cakmak et al., 2010). In Nepal, the germplasm of durum wheat developed in CIMMYT Mexico was being introduced regularly at the National Wheat Research Program, Bhairahawa, but could not advance further due to poor performance in that location. Therefore, evaluation was planned in western plain Terai. For this, the Agriculture Botany Division introduced 55 selected lines from a small plot (PC) trial from CIMMYT, El Batan, Mexico in 2007 targeting mid and far-western plain area (Terai), and tested at Regional Agriculture Research Station, NARC, Khajura, Banke and twenty lines were selected for their acceptable resistance to powdery mildew. Further introduction of International Durum Wheat Screening Nursery (IDSN) and International Durum Wheat Yield Nursery (IDYN) from CIMMYT, Mexico started regularly. It tested and selected superior lines for further yield trials. The first Durum Wheat Advanced Varietal Trial (DWAVT) was formed in the 2011/12 season and is being continued to identify superior genotypes suitable for the industrial production of semolina and pasta in Nepal. The current research involved i) testing of genotypes selected from CIMMYT’s IDSN, and IDYN nurseries; ii) composing durum wheat advanced varietal trials (DWAVT) for at least two years; iii) testing of best lines in Participatory Variety Selection (PVS) trials for verification at farmers field, and iv) variety release. So far, only two varieties of durum wheat namely Khajura Durum 1 and Khajura Durum 2 have been released in Nepal (Nepal Rajpatra, 2018).

In this paper, we conducted a comprehensive evaluation of different durum wheat lines developed by CIMMYT, including the first two varieties released in Nepal for grain yield and several agronomic traits for 10 years at the national research center, with the aim of selecting some more superior performing durum wheat lines for advancement and release in the country. This is the first study of its kind to systematically assess the grain yield and associated traits of durum wheat across multiple environments in Nepal, providing a long-term perspective on genotype performance. Over the past decade, Nepal has experienced a range of climatic challenges that have significantly impacted agricultural productivity, including increased temperature fluctuations, unpredictable monsoon patterns, and prolonged droughts. These environmental stressors have heightened the need for developing resilient durum wheat varieties capable of withstanding such conditions, making this long-term study particularly relevant. The results of this study contribute significantly to the wheat community by offering insights into the genetic diversity and adaptability of durum wheat in South Asia. The identification of promising genotypes with stable performance across diverse environments will be invaluable for breeders aiming to develop new varieties with enhanced yield potential and resilience. Furthermore, our findings provide a foundation for future research on the underlying genetic mechanisms driving these traits, thereby supporting the development of durum wheat varieties that are better equipped to meet the challenges of climate change and food security. This manuscript adds to the wheat community by filling a critical gap in the literature on durum wheat adaptation and performance in Nepal. Our research not only highlights the potential of certain genotypes for breeding programs but also emphasizes the importance of long-term, multi-environment trials in understanding genotype-environment interactions. By doing so, this work supports the broader goals of improving wheat productivity and sustainability in challenging agricultural regions.

## 2 Materials and methods

### 2.1 Plant materials

This study includes elite durum wheat lines developed at CIMMYT, Mexico, and introduced in Nepal for testing and advancement in yield trials. The introduced durum wheat lines from DWON, 40^th^ to 50^th^ International Durum wheat Screening Nurseries (40^th^-50^th^ IDSN) and 42^nd^ to 51^st^ International Durum Wheat Yield Nurseries (42^nd^-51^st^ IDYN) were tested and best performing lines were selected to constitute DWAVT at Regional Agriculture Research Station, Khajura, Banke, Nepal as a part of the ongoing wheat breeding activity. This paper reports the observations collected from 10 cycles of DWAVT trials (1^st^ to 10^th^ DWAVT) from 10 years (2011/12 to 2020/21). Each of these DWAVTs consisted of thirty durum wheat genotypes, including 26 selected genotypes and four international checks, namely Altar 84, Jupare C2001, Mexicali C 75, Yavaros 79. We used the same check varieties till 2017/18. After 2017/18, we added Khajura Durum 1 and Khajura Durum 2 and only two from international checks for the trials. Altogether, one hundred and thirty-two genotypes, including four international standard checks, Altar84, Jupare C2001, Mexicali C75, and Yavaros79, were evaluated over ten years (**Supplementary Table S1**).

### 2.2 Experimental sites

**Figure 1:**
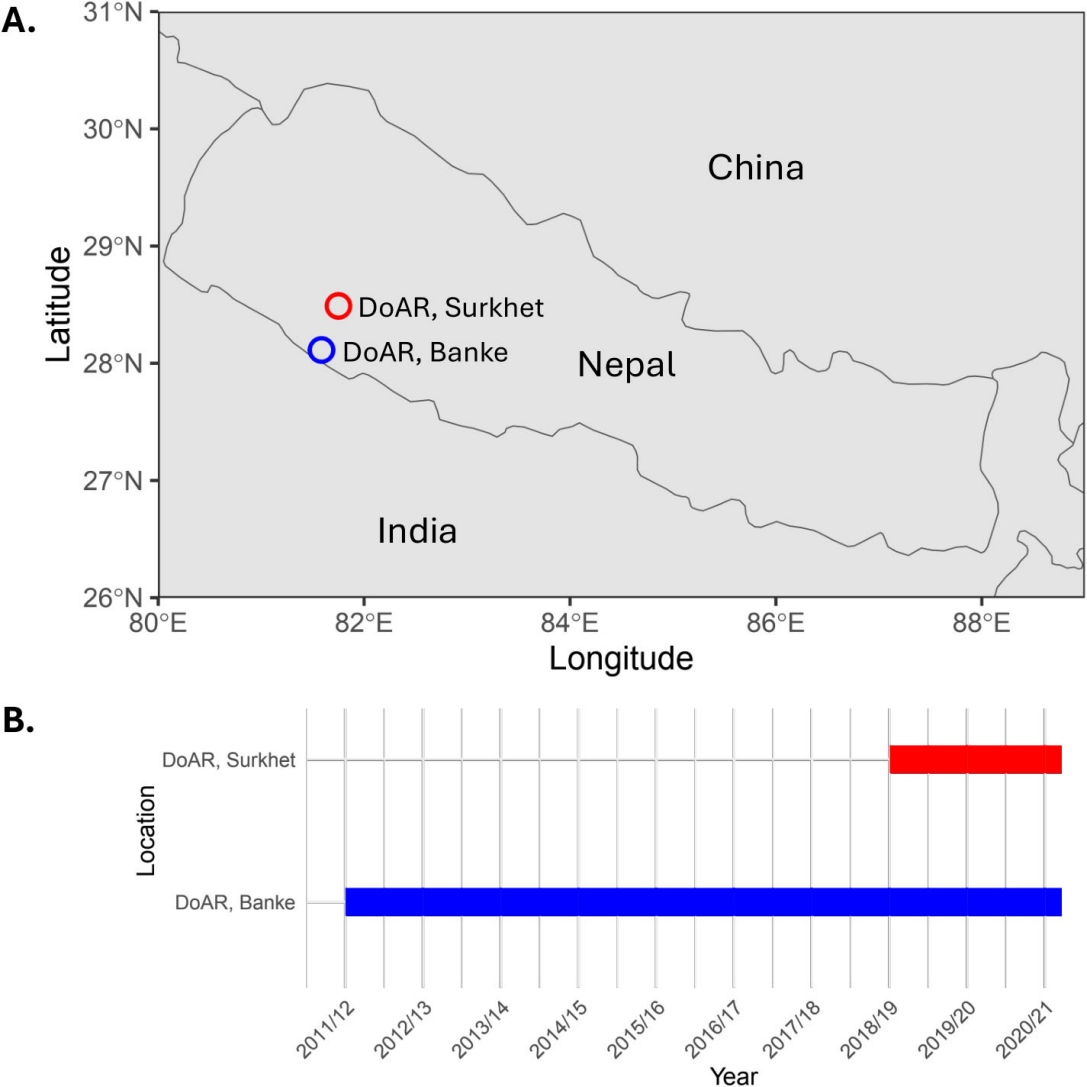
Location and duration of the trials conducted in this study. **(A)** Map showing the location of the research stations where the trials were conducted. **(B)** Timeline graph showing the time periods of trials conducted.

The field screening and testing of trials (1^st^ DWAVT to 10^th^ DWAVT) were conducted at the Directorate of Agriculture Research (DoAR), NARC, Khajura, Banke (formerly, Regional Agricultural Research Station, NARC, Khajura, Banke). The station lies at 28° 06’ N latitude 81° 37’ E longitude, with an altitude of 181 meters above sea level. The soil texture is sandy loam with a pH value of 7.3. In addition to the Khajura site, the 8^th^ DWAVT to 10^th^ DWAVT were tested at the Directorate of Agriculture Research, NARC, Dashrathpur, Surkhet (formerly, Agricultural Research Station, Dasharathpur, Surkhet) which is located at 28^0^29’21” N latitude, 81^0^45’16” E longitude with the elevation of 501m asl (**Figure 1**). The soil texture is sandy loam with a pH value of 6.6.

### 2.3 Experimental design, trial management, and data collection

Each trial was performed under the Alpha Lattice design with three replications. Plot size was 6 m^2^ (3m length and 2m breadth). Inorganic fertilizers were applied on split doses at the rate of 60:60:60 NPK kg/ha at land preparation, 30:00:00 NPK kg/ha at tillering, and 30:00:00 NPK kg/ha at booting stage. Three irrigations were given during the stages of pre-sowing, tillering (25 days after sowing), and booting (55 days after sowing). The genotypes were evaluated for 8 phenological, agro-morphological, grain yield, and its attributes. Days to heading (DH) were recorded when 50% of plants in a plot had extended spike from the leaf sheath. Days to maturity (DM) were recorded when 80% of the plants in a plot had peduncles that had turned from green to golden yellow. Plant height (PHT) was measured as the length from ground level to the apex of the spike excluding awns. For grain number per spike (GNS) and grain weight per spike (GWS), we sampled 10 spikes per plot, threshed them, counted them manually, weighed, and then averaged them for GNS and GWS. The total heads per square meter was assessed by measuring the number of tillers counted and converted into the number of heads per square meter. Spike length was measured on a representative spike within the plot as the length from the base to the tip of a spike excluding awns. Peduncle length was measured on representative plants within the plot as the length from the flag leaf to the base of a spike. Diseases were observed sporadically in some years in DWAVT, specifically during 2012/13, 2017/18, 2019/20 and 2020/21, in field conditions at Khajura to assess the disease response in western terai condition. Disease assessments were conducted after anthesis. Foliar blight disease severity was evaluated using the modified Saari and Prescott’s double-digit scale (00 to 99) (Eyal, 1987). Similarly, leaf rust was scored using a modified Cobb scale (Peterson et al., 1948). Further description of the data collection is provided in **Supplementary Table S2**.

### 2.4 Statistical analysis

Analysis of variance and multiple comparison tests was performed using the “agricolae” package in R (de Mendiburu and de Mendiburu, 2019). The overall datasets across 10 years were also analyzed using Best Linear Unbiased Prediction (BLUP) based on the variance component in the mixed model as done previously (Thapa et al., 2022). For this, we used only eight traits recorded throughout the years (**Supplementary Table S3**). In the model, the genotype effects, block, and location were all set as random. The cluster dendrograms were constructed using the ggtree package in R (Yu et al., 2017). Estimated BLUP values of eight traits were used for cluster analysis. These traits were selected because they were consistently recorded and contributed significantly to the major principal components (**Supplementary Table S4**). Principal Component Analysis (PCA) and eigenvalue calculations were performed using the “FactoMineR” package in R (Lê et al., 2008). Cluster analysis was performed as previously done (Thapa et al., 2022). Correlation analysis was performed by calculating Pearson’s correlation coefficient 125 using a ggcorrplot package in R (Kassambara and Kassambara, 2019). The correlation network was constructed using the “igraph” package in R (Csardi and Nepusz, 2006). Broad-sense heritability (H2) for all the studied traits was computed according to Allard (1999). Expected genetic advance (GA), assuming a selection intensity of 5% was calculated following Allard (1999). All the statistical analyses otherwise mentioned were performed in R (R Core Team, 2021). Map of study locations was generated using R package “rnaturalearth” (South, 2017; https://CRAN.R-project.org/package=rnaturalearth) and “ggspatial” (Dunnington, 2023; https://CRAN.R-project.org/package=ggspatial) as described previously (Sapkota et al., 2023; Zhao et al., 2024). The package “rnaturalearth” uses the public domain map dataset “Natural Earth” (https://www.naturalearthdata.com/) to generate the base layer of the map. The timeline plot was created using “ggplot2” package in R (Wickham, 2011).

### 2.5 Hypotheses tested

Different statistical analyses were performed to test several hypotheses focused on the performance of durum wheat genotypes across multiple environments and the association between specific agronomic traits and yield. The first hypothesis assumes that the durum wheat genotypes exhibit significant variability in grain yield and associated agronomic traits across different environments in Nepal. The null hypothesis asserts no difference in the means of the studied traits among the genotypes, while the alternate hypothesis suggests a difference exists, reflecting the diverse genetic backgrounds and their varying performance under distinct environmental conditions, thereby aiding in the identification of high-yielding and stable genotypes. The second hypothesis explores the correlation among the different agronomic traits studied. Here, the null hypothesis proposes no correlation, whereas the alternate hypothesis suggests the presence of correlations, based on the expectation that these traits might be physiologically linked or share common pathways. Understanding these relationships could assist breeders in selecting or studying these traits in conjunction. The third hypothesis suggests that the studied traits will enhance our understanding of genotypic performance and are crucial for clustering, which helps identify patterns, relationships, and groups of genotypes with similar features, from which breeders can select for crossing or breeding purposes. The null hypothesis for this scenario asserts that all genotypes are similar, with no clusters present, while the alternate hypothesis suggests the existence of distinct clusters or groups of genotypes with similar traits. These hypotheses were important to our experimental design and analysis, ensuring that the study addresses key questions related to genotype performance, trait variability, and the potential for selecting superior durum wheat genotypes for cultivation in Nepal.

## 3 Results

### 3.1 Variation in yield and associated traits evaluated over ten years

A total of 132 durum wheat genotypes were cultivated and assessed over a span of ten years (**Supplementary Tables S3, S5**). The BLUP analysis results (**Supplementary Table S3**) for DWAVT conducted from 2011-12 to 2020-21 revealed a highly significant variance among the tested genotypes for all the studied traits, including grain yield (**Table 1**).

**Table 1:** Top ten high yielding durum wheat genotypes and checks among 132 durum wheat genotypes tested in DWAVT from 2011/12 to 2020/21.

SN	Entry	DH	DM	PHT	TN	GNS	GWS	TGW	GY	GY_Rank_
**1**	**DWK38**	88.20	125.05	91.98	295.79	52.20	2.31	44.29	4416.04	**1**
2	DWK139	89.08	126.13	90.15	325.46	48.51	2.22	44.65	4406.10	2
3	DWK125	87.83	124.52	91.69	309.78	49.80	2.12	42.83	4353.50	3
4	DWK120	87.86	125.11	92.12	305.16	52.34	2.24	42.98	4351.15	4
5	DWK143	90.18	125.82	91.29	318.04	48.23	2.16	44.81	4338.82	5
6	DWK98	89.32	125.45	89.85	308.90	50.35	2.12	41.76	4333.24	6
7	DWK117	88.98	124.31	87.49	322.33	47.51	2.07	43.05	4332.66	7
8	DWK134	87.79	125.86	92.38	298.66	45.55	2.06	44.32	4307.96	8
9	DWK94	90.52	125.65	92.52	298.69	50.17	2.22	44.01	4299.40	9
10	DWK118	88.71	126.12	93.20	308.66	50.02	2.22	44.53	4292.80	10
**11**	**ALTAR84**	86.56	123.52	87.56	304.97	50.83	2.04	39.65	4282.37	**12**
12	**JUPAREC2001**	89.40	126.08	90.82	298.11	52.46	2.17	41.28	4040.92	**125**
13	**MEXICALIC75**	89.70	125.84	89.05	285.47	45.27	2.07	44.54	3920.55	**131**
14	**YAVAROS79**	84.12	122.60	87.89	278.91	47.24	2.09	43.50	3908.06	**132**
	**Mean**	**88.29**	**125.28**	**89.47**	**302.66**	**49.14**	**2.13**	**42.93**	**4155.19**	
	**SD**	1.67	0.82	2.32	9.78	1.51	0.06	1.20	85.92	
	**CV (%)**	1.89	0.66	2.59	3.23	3.06	2.65	2.80	2.07	
	**P-value**	0	0	0	0	0	0	0	0.004	

DH, Days to heading; DM, Days to maturity; PHT, Plant height (cm); TN, number of tillers per square meter; GNS, Grain number per spike; GWS, Grain weight per spike (gm); TGW, Thousand grain weight(gm) and GY, Grain yield (kg/ha).

A combined analysis of variance was performed for all 132 durum wheat genotypes across ten years and two locations (last two years) and the trait means in the table are BLUP values for corresponding studied traits. The bold entry names indicate the checks and one of the varieties released DWK38.

The heading and maturity of the durum wheat genotypes varied significantly, with Yavaros 79 and DWK75 being the earliest in terms of heading (84 days), and DWK75 being the earliest in maturity (122 days). On the other hand, DWK101 and DWK119 took the longest time to reach heading (92 days), while DWK81 took the longest time to attain physiological maturity (128 days). Additionally, DWK141 and DWK137 exhibited the shortest plant stature (83 cm), while DWK47 and DWK84 were the tallest (94 cm). Similarly, DWK90 had the highest tiller numbers (333 per sq. m), followed by DWK142 (330 per sq. m) and DWK91 (327 per sq. m).

The per spike grain numbers were highest in the check varieties Jupare C 2001, DWK27, Khajura Durum 2 (DWK38), DWK77, DWK76, DWK88, DWK120, and DWK147, with 52 grains per spike. Furthermore, DWK38 exhibited the highest grain weight per spike (2.3 g). However, DWK110 showcased the boldest seeds, with a thousand grain weight of 47.2 g, followed by DWK135 (47.1 g) and DWK103 (46.1 g).

The grain yield (BLUP) ranged from 3908 kg/ha (Yavaros 79) to 4416 kg/ha (DWK38), with a mean gain of 4155 kg/ha. Among the four check varieties, Altar 84 ranked 12th with a grain yield of 4282 kg/ha, indicating its superior performance. Jupare C 2001 (4041 kg/ha), Mexicali C 75 (3921 kg/ha), and Yavaros 79 (3908 kg/ha) ranked 125th, 131st, and 132nd, respectively. The highest yielding genotypes were DWK38 (4416 kg/ha), followed by DWK139 (4406 kg/ha) and DWK125 (4354 kg/ha) (**Table 1**). Notably, DWK38 held the top rank for grain yield for three years and remained among the top ten genotypes for seven out of the ten years tested in DWAVT (**Table 2**).

**Table 2:** Comparison of DWK38 and DWK26 with best checks based on grain yield(GY in kg/ha) on DWAVT from 2011/12 to 2020/21 in Nepal.

	DWK38		DWK26		Best check		
Year	GY	Rank	GY	Rank	GY	Rank	Genotype
2011-12	6722	1	6222	3	5861	8	ALTAR 84
2012-13	3765	4	3428	9	3315	13	ALTAR 84
2013-14	5172	3	4885	11	5115	5	JUPARE C 2001
2014-15	5399	1	4914	4	4285	16	ALTAR 84
2015-16	3157	18	3626	6	2773	24	ALTAR 84
2016-17	4067	23	3876	29	4926	10	ALTAR 84
2017-18	3570	20	2728	27	3872	10	ALTAR 84
2018-19	4259	10	2944	28	4772	2	ALTAR 84
2019-20	3259	1	2329	27	2430	25	ALTAR 84
2020-21	4489	3	4194	9	4136	12	YAVAROS 79
Mean	4386	8	3914	15	4149	13	

### 3.2 Disease evaluation

There was no uniformity of disease occurrence in the tested years. We did not observe any yellow rust and stem rust in any of the tested years, whereas *Helminthosporium* leaf foliar blight (HLB) was observed in three growing seasons (2017/18, 2019/20 and 2020/21). Leaf rust was observed only in 2017/18. The results revealed that most genotypes had the lowest leaf rust disease severity during 2017/18. Among the tested 30 genotypes only 7 genotypes (DWK141, DWK109, DWK144, DWK125, DWK120, DWK133, and DWK135) scored 5R and 3 genotypes (DWK135, DWK130, and DWK131) scored 10 R for leaf rust and remaining all the genotypes were immune to leaf rust and exhibited no symptoms.

During 2012/13, a heavy incidence of Rhizoctonia infestation on tested genotypes ranged from 1 to 47% severity. Genotypes with the least infestation were DWK46, DWK81 (1%), DWK 28 (7%), DWK72, DWK82 (8%), DWK67 and Mexicali C75(10%). Among tested genotypes, DWK13 and Yavaros79 had the highest severity, with 47 and 40%, respectively. Less than 10% infection was observed among the 7 genotypes (DWK46, DWK81, DWK82, DWK28, DWK 67, MEXICALI 75 and DWK72) categorized as a resistant category. Twelve genotypes (DKW30, DKW38, DKW47, DKW55, DKW61, DKW73, DKW75, DKW76, DKW77, DKW78, DKW79 and DKW80) were categorized in the moderate resistant category with 11-20% *Rhizoctinia* blight infection. Likewise, 5 genotypes (Altar84, DWK41, DWK29, DWK83 and JUPARE C2001) with 21-30% *Rhizoctonia* blight infection were categorized as moderate susceptible. Five genotypes (DWK11, DWL2, DWK26, DWK74 and YAVAROS79) with 31-40% infection were classified as susceptible. One genotype DWK13 with 43% *Rhizoctonia* blight infection was highly susceptible (**Table 3**).

**Table 3:** Disease severity observed for Rhizoctonia infection in 2012/13 and HLB in 2017/18, 2019/20 and 2020/21 in different durum wheat genotypes.

2012/13		2017/18		2019/20		2020/21	
Genotype	Rhi Inf	Genotypes	HLB severity %	Genotypes	HLB severity %	Genotypes	HLB severity %
ALTAR 84	23.6	ALTAR 84	7.4	ALTAR 84	13.1	ALTAR 84	44.4
DWK11	33.3	DWK102	10.2	DWK 121	3.7	DWK 110	11.1
DWK13	46.6	DWK106	7.4	DWK 145	3.7	DWK 116	11.1
DWK2	33.3	DWK109	6.5	DWK 146	12.3	DWK 121	11.1
DWK26	31.6	DWK110	7.4	DWK 147	18.5	DWK 134	11.1
DWK28	7.0	DWK116	2.8	DWK110	2.4	DWK 138	22.2
DWK29	30.0	DWK117	3.7	DWK116	8.2	DWK 145	11.1
DWK30	11.6	DWK118	7.4	DWK120	3.7	DWK 147	11.1
DWK38	18.3	DWK120	10.8	DWK134	8.6	DWK 151	11.1
DWK41	26.6	DWK121	6.8	DWK138	3.7	DWK 152	11.1
DWK46	1.3	DWK124	2.8	DWK139	4.9	DWK 153	22.2
DWK47	17.6	DWK125	7.4	DWK144	4.9	DWK 154	22.2
DWK55	20.0	DWK126	7.4	DWK149	9.8	DWK 156	33.3
DWK61	17.6	DWK128	3.7	DWK150	18.1	DWK 157	11.1
DWK67	10.3	DWK130	3.7	DWK151	12.7	DWK 158	11.1
DWK72	8.3	DWK131	7.4	DWK152	6.1	DWK 159	44.4
DWK73	15.0	DWK133	11.1	DWK153	4.9	DWK 164	22.2
DWK74	36.6	DWK134	7.4	DWK154	9.8	DWK 165	22.2
DWK75	16.6	DWK135	10.8	DWK155	13.5	DWK 166	11.1
DWK76	11.6	DWK137	7.4	DWK156	7.0	DWK 167	22.2
DWK77	15.0	DWK138	2.8	DWK157	2.4	DWK 168	22.2
DWK78	13.6	DWK139	7.4	DWK158	11.1	DWK 169	11.1
DWK79	20.0	DWK140	6.5	DWK159	11.1	DWK 170	11.1
DWK80	12.0	DWK141	6.5	DWK160	12.3	DWK 171	33.3
DWK81	1.0	DWK142	11.1	DWK161	18.5	DWK 172	22.2
DWK82	8.3	DWK143	6.8	DWK162	2.4	DWK 173	22.2
DWK83	23.3	DWK144	3.7	DWK163	13.1	DWK 174	11.1
JUPARE C 2001	26.6	Khajura Durum 1(=DWK26)	6.8	Khajura Durum 1 (=DWK26)	10.6	Khajura Durum 1 (=DWK26)	22.2
MEXICALI 75	16.6	Khajura Durum 2(=DWK38)	6.8	Khajura Durum 2 (=DWK38)	3.7	Khajura Durum 2 (=DWK38)	11.1
YAVAROS 79	40.0	YAVAROS 79	3.7	YAVAROS 79	13.2	YAVAROS 79	33.3

HLB disease scoring was observed in experiment trials tested in Khajura, Banke in 2017/18, 2019/20 and 2020/21. Double digit scoring was carried out and converted into percentage digit severity to quantify the response of durum wheat genotypes to HLB disease. In 2017/18, the average disease severity was 6.67% with maximum disease severity of 15%. Standard check Khajura Durum 1 and Khajura Durum 2 also possessed less than 10% disease severity. DWK116, DWK117, DWK124, DWK128, DWK130, DWK138, and DWK144 had less than 5% HLB severity (**Table 3**). In the year 2019/20, the Average HLB disease severity was 8.96% with a range of 2.46 to 18.51%. DWK147, DWK150, and DWK161 ranged between 15 to 20%, but many tested genotypes had less than 15% of HLB severity (**Table 3**). In 2020/21, the average disease severity was 19.33%, ranging from 11.1% to 44.4%. Highest disease severity (44.44%) was observed in Altar84 and DWK159 respectively. DWK38 possessed 11.1% disease severity, which was lower than the grand mean disease severity. Highest severity was observed on Altar84 and DWK159 (44.4%), followed by DWK156, DWK176, Yavaros79 (33.3%), DWK138, DWK153, DWK154, DWK172, DWK173 and DWK138 (22.2%). The remaining genotypes had severity of 11.1% (**Table 3**).

### 3.3 Phenotypic correlation

**Figure 2:**
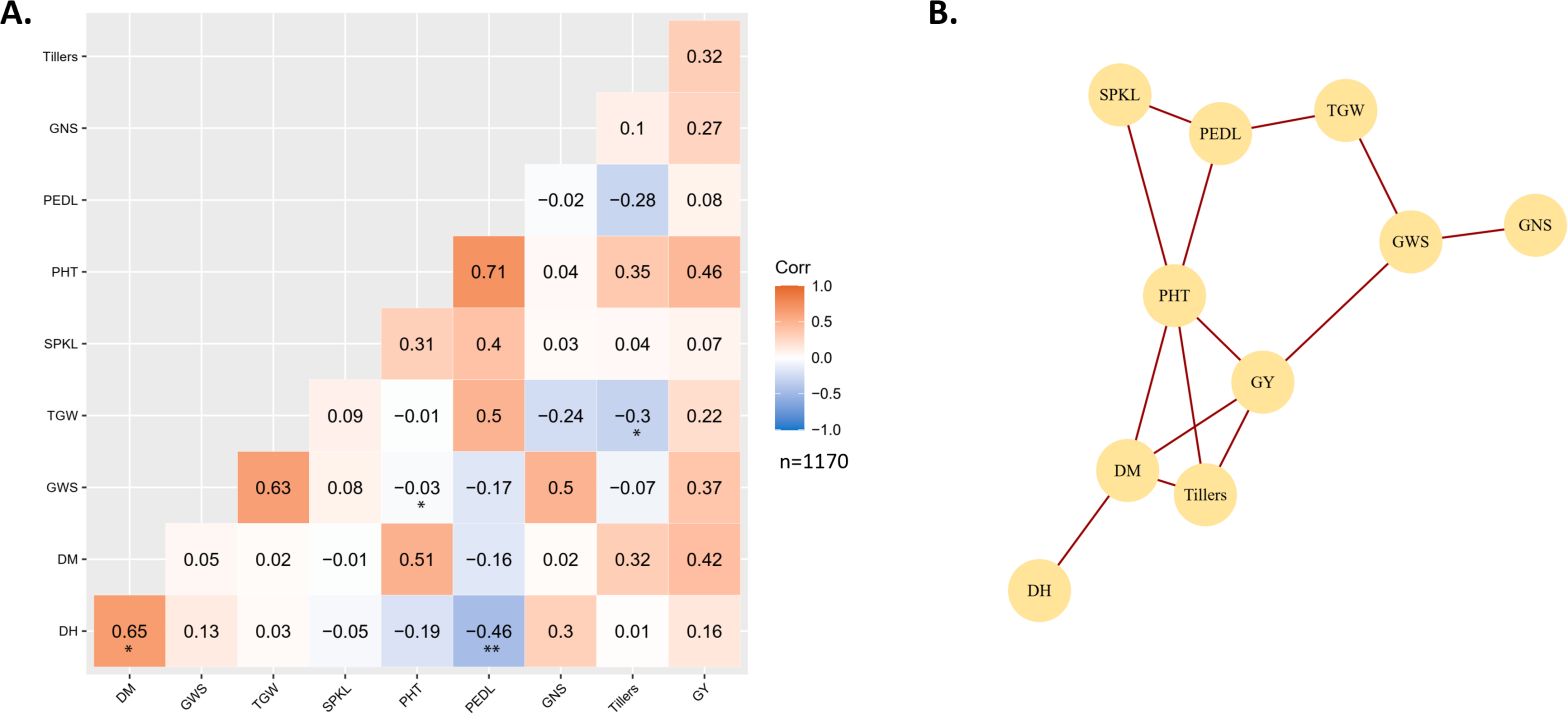
Phenotypic correlation between grain yield and other phenological and yield attributing traits of 132 durum wheat genotypes tested from 2011/12 to 2020/21 in Nepal. **(A)** Heatmap representing phenotypic correlation matrix of all the studied traits. The significance levels below the correlation coefficient are *: p < 0.05, **: p < 0.01 and ***: p < 0.001. **(B)** Correlation network representing highly associated traits after correlation analysis.

Pearson correlation coefficients were computed for the major phenological, and yield traits of the 132 durum wheat genotypes tested during the 2011/12 to 2020/21 season (**Figure 2A**). A significant and strong correlation was observed between heading and maturity days (r=0.65*). The days to heading were negatively and significantly associated with peduncle length (r=-0.46**). Maturity days strongly correlated with the plant height (r=0.51) and grain yield (0.42), but the association was not statistically significant. Thousand grain weight was negatively and significantly correlated with tiller numbers (r=-0.3*) but positively correlated with peduncle length (r=0.5). However, the later association was not significant. Likewise, grain weight per spike strongly correlates with thousand grain weight (r=0.63) and grain number per spike (r=0.5). Grain yield was also found to be positively correlated with plant height (r=0.46), tiller numbers (r=0.32) and grain weight per spike (r=0.37). The correlation network showed that days to maturity, plant height, tiller numbers, and grain weight per spike were directly associated with grain yield (**Figure 2B**).

### 3.4 Heritability

We computed heritability and genetic advancement of means for all the trials to investigate the genetics of the traits evaluated (**Supplementary Table S6**). Average heritability calculated throughout the years showed that days to heading (0.89), days to maturity (0.81), plant height (0.76), spike length (0.68), peduncle length (0.75), tillers number per square meter (0.73), and grain weight per spike (0.65) were highly heritable. Grain number per spike (0.47), thousand grain weight (0.52), and grain yield (0.50) were found to have moderate heritability. We also observed high genetic advancement of the mean for spike length (22.72%), peduncle length (27.92%), tiller numbers (37.20%), grain weight per spike (31.36%), and grain yield (23.53%). In contrast, we observed moderate genetic advancement of the mean for plant height (13.93%), and thousand grain weight (17.94%) (**Table 4**).

**Table 4:** Heritability and genetic advance of studied traits of DWAVTs 2011/12–2020/21 seasons.

Parameter	DH	DM	PHT	SPK L	Ped L	Tillers/m2	GNS	GWS	TGW	GY
Heritability (%)	0.89	0.81	0.76	0.68	0.75	0.73	0.47	0.65	0.52	0.50
GA	8.80	7.70	11.11	1.68	4.67	111.84	5.24	0.62	7.18	871.83
GAM (%)	10.08	6.17	13.93	22.72	27.92	37.20	8.79	31.36	17.94	23.53

DH, days to heading; DM, days to maturity; PHT, plant height; SPK L, spike length; Ped L, peduncle length; GNS, grain number per spike; GWS, grain weight per spike; TGW, thousand grain weight; GY, grain yield.

### 3.5 Cluster analysis

**Figure 3:**
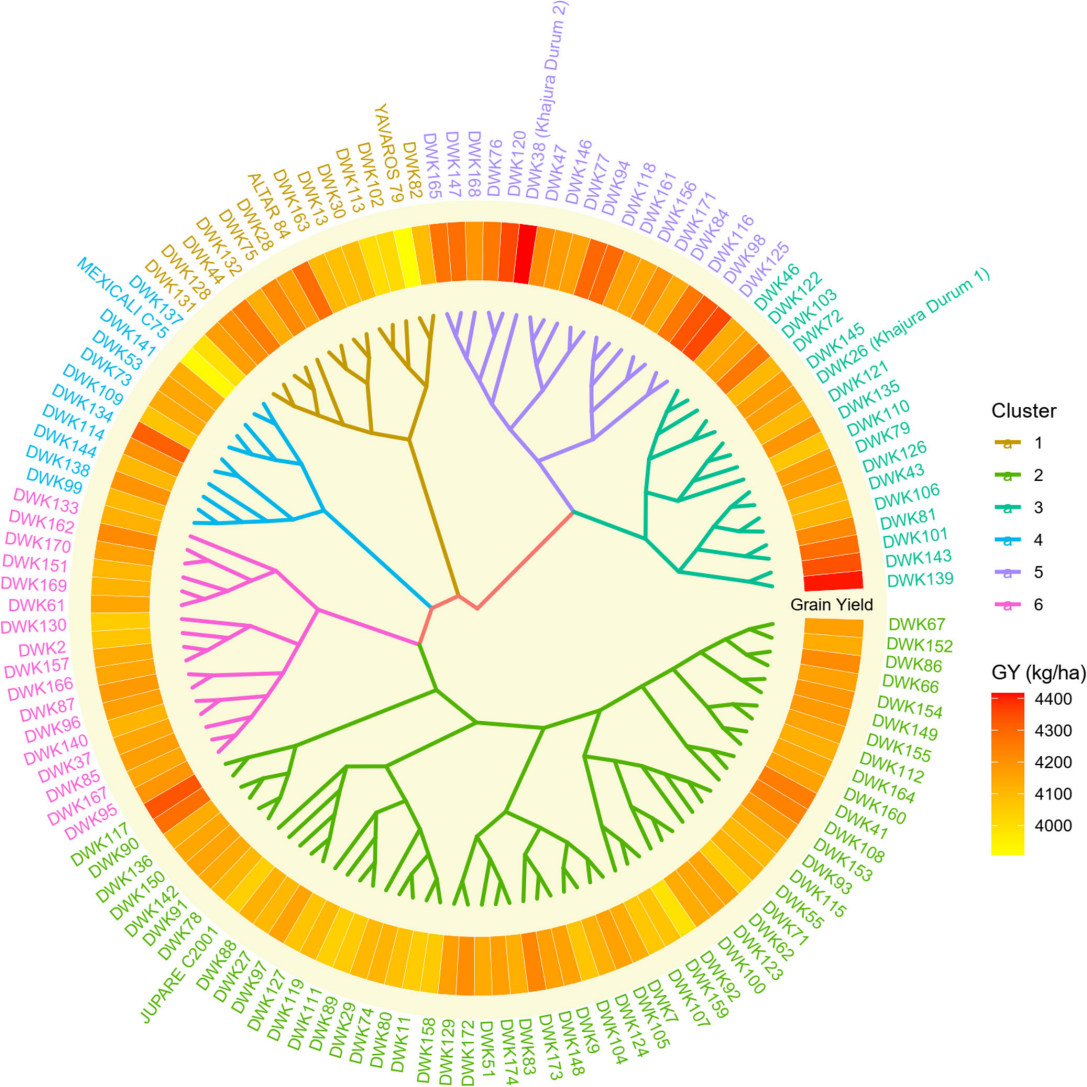
Clustering of all durum wheat genotypes from 1^st^ to 10^th^ DWAVT based on BLUP values.

Principal Component Analysis (PCA) was conducted, and eigenvalues were calculated for each component. The first four components, each with an eigenvalue greater than one, accounted for approximately 82.12% of the total variance, indicating that they capture the majority of the dataset’s information (**Supplementary Table S4**). The PCA loadings revealed that all eight traits analyzed significantly contribute to one or more of these principal components (**Supplementary Table S4**). Consequently, all eight traits were utilized for cluster analysis to identify genotype clusters with specific agronomic characteristics. Cluster analysis performed using the BLUP estimates resulted in six distinct clusters based on days to heading, days to maturity, plant height, tillers number, grain number per spike, grain weight per spike, thousand grain weight, and grain yield (**Figure 3**). Cluster 1, 2, 3, 4, 5, and 6 had 14, 55, 17, 11, 18, and 17 genotypes respectively (**Table 5**). Genotypes belonging to cluster 1 had concise heading (85.50 days) and maturity days (123.72 days) and had low yield too (4121.66 kg/ha). The checks ALTAR84 and YAVAROS79 belonged to this cluster. Cluster 2 had the lowest thousand grain weight (42.34 gm) with the longest heading days (89.19 days), including one of the checks, JUPAREC2001. Cluster 3 had accessions with the highest thousand gain weight (44.79 gm), most tillers (307.85), and second highest grain yield (4184.97 kg/ha) and plant height (91.22 cm). However, this cluster took the longest to mature. DWK26 belonged to cluster 3. Cluster 4 had the lowest yield (4118.06 kg/ha), grain weight per spike (2.07 gm), tillers (293.27), and grain number per spike (46.85). The next cluster, cluster 5 exhibited the highest grain yield (4251.18 kg/ha), grain number per spike (50.78), and plant height (91.77 cm) and included high yielding genotypes like DWK38. Cluster 6 had the shortest plant height (87.23 cm) and had average values for other traits studied (**Table 5**).

**Table 5:** Cluster mean of genotypes clusters obtained from the analysis of durum wheat genotypes.

Cluster	No. of genotypes	DH	DM	PHT	TILLERS	GNS	GWS	TGW	GY	Genotypes
1	14	85.50	123.72	89.71	297.54	49.02	2.12	42.59	4121.66	ALTAR84, DWK102, DWK113, DWK128, DWK13, DWK131, DWK132, DWK163, DWK28, DWK30, DWK44, DWK75, DWK82, YAVAROS79
2	55	89.19	125.58	89.10	305.28	49.18	2.11	42.34	4134.97	DWK100, DWK104, DWK105, DWK107, DWK108, DWK11, DWK111, DWK112, DWK115, DWK117, DWK119, DWK123, DWK124, DWK127, DWK129, DWK136, DWK142, DWK148, DWK149, DWK150, DWK152, DWK153, DWK154, DWK155, DWK158, DWK159, DWK160, DWK164, DWK172, DWK173, DWK174, DWK27, DWK29, DWK41, DWK51, DWK55, DWK62, DWK66, DWK67, DWK7, DWK71, DWK74, DWK78, DWK80, DWK83, DWK86, DWK88, DWK89, DWK9, DWK90, DWK91, DWK92, DWK93, DWK97, JUPAREC2001
3	17	88.95	125.91	91.22	307.85	48.43	2.18	44.79	4184.97	DWK101, DWK103, DWK106, DWK110, DWK121, DWK122, DWK126, DWK135, DWK139, DWK143, DWK145, DWK26 (Khajura Durum 1), DWK43, DWK46, DWK72, DWK79, DWK81
4	11	87.88	125.69	87.98	293.27	46.85	2.07	43.29	4118.06	DWK109, DWK114, DWK134, DWK137, DWK138, DWK141, DWK144, DWK53, DWK73, DWK99, MEXICALIC75
5	18	88.07	125.02	91.77	301.97	50.78	2.20	43.22	4251.18	DWK116, DWK118, DWK120, DWK125, DWK146, DWK147, DWK156, DWK161, DWK165, DWK168, DWK171, DWK38 (Khajura Durum 2), DWK47, DWK76, DWK77, DWK84, DWK94, DWK98
6	17	87.51	125.02	87.23	300.25	50.14	2.16	42.70	4140.83	DWK130, DWK133, DWK140, DWK151, DWK157, DWK162, DWK166, DWK167, DWK169, DWK170, DWK2, DWK37, DWK61, DWK85, DWK87, DWK95, DWK96

DH, Days to heading; DM, Days to maturity; PHT, Plant height (cm); TILLERS, number of tillers per square meter; GNS, Grain number per spike; GWS, Grain weight per spike (gm); TGW, Thousand grain weight(gm) and GY, Grain yield (kg/ha).

### 3.6 Breeder seed production and distribution of durum wheat varieties in Nepal

Recognizing the importance of domestic production of durum wheat, two varieties, namely DWK26 (Khajura Durum 1) and DWK38 (Khajura Durum 2), belonging to clusters 3 and 5, respectively, were selected and released as the first durum wheat varieties in Nepal in 2017. The Nepal Agricultural Research Council (NARC) undertook rigorous research for ten years, including on-station (**Tables 1**, **2**; **Supplementary Tables S3, S5**) and on-farm trials, to ensure the acceptance of these varieties among farmers. Further evaluations were conducted on other promising genotypes, focusing on grain yield and quality, to select the best varieties for future release. The production of source seeds for released and pre-release durum wheat varieties gained momentum in Nepal through the involvement of NARC and farmers (**Table 6**).

**Table 6:** Breeders seed production and distribution status of DoAR, khajura.

Fiscal Year	Khajura Durum 1 (kg)	Khajura Durum 2 (kg)
2016/17	340	500
2017/18	200	350
2018/19	720	750
2019/20	266	387
2020/21	190	290
2021/22	0	72
2022/23	150	300
Total	1866	2649
	Price per kg (NPR)	65

NPR, Nepalese Rupees.

During the 2018/19 season, durum wheat cultivation covered an estimated area of 800 hectares. By 2021/22, NARC had produced 4 Quintals of breeder seed for durum wheat, which included Khajura Durum 1 and Khajura Durum 2. As per Timsina et al. (2018), one ton of breeder seed and 15 Mt of foundation seed is sufficient to meet the seed requirements for 5% of Nepal’s wheat area (36,000 ha). This can be achieved through regular seed multiplication per the prescribed seed multiplication ratio and seed cycle (from breeder to foundation, and from foundation to certified seeds - C1 and C2), provided proper planning and coordination exists.

Overall, establishing breeder seed production and distribution programs for durum wheat varieties in Nepal demonstrates the country’s commitment to self-sufficiency and sustainable agricultural practices.

## 4 Discussion

Crop improvement relies on high variation within crop resources, allowing plant breeders to create novel combinations of plant genes and select crop varieties better suited for diverse agricultural systems (Bhandari et al., 2017). In our study, we observed significant variation among the durum wheat genotypes for grain yield and associated traits, demonstrating the wide range of phenotypic and genotypic diversity present in the accessions introduced in Nepal from CIMMYT lines. This high variation is essential for achieving greater genetic gains in breeding programs. Furthermore, observing disease resistance and its variation across different years highlights the importance of these genotypes for breeding disease-resistant varieties.

Multiyear evaluations are crucial for identifying stable, high-yielding lines that perform consistently well in breeding programs, including durum wheat breeding. Testing and assessment are vital in introducing a new crop in any country. Our study represents the first comprehensive evaluation of yield and associated traits for durum wheat lines introduced and studied in Nepal. Using a fast-track approach, we conducted these trials and tests over ten years to identify stable and high-yielding durum wheat genotypes suitable for Nepal. These trials and tests played a pivotal role in releasing the country’s first two durum wheat varieties. Our findings not only highlight the variability and adaptability of durum wheat genotypes across different environments in Nepal but also provide key insights into the traits that are most critical for breeding programs. By identifying genotypes with superior performance under diverse climatic conditions, this study offers a roadmap for future breeding strategies that aim to enhance durum wheat production in Nepal. These results will aid in developing varieties that are more resilient to the challenges posed by climate change, thereby ensuring food security and sustainable agricultural practices in the region.

The heritability estimates for the same crop and trait can vary widely due to differences in experimental materials and setups (Garcia-Oliveira et al., 2009). Selection efficiency is influenced by the magnitude of heritability and genetic advance (Singh and Singh, 2015). According to the heritability categories defined by Comstock et al. (1949), traits such as days to heading and maturity, plant height, spike length, peduncle length, tillers per unit area, and grain weight per spike exhibited high heritability, indicating their ability to transmit to the next generation. Meanwhile, grain number per spike, thousand grain weight, and grain yield showed moderate heritability. High broad-sense heritability provides insight into the total variation ascribable to genotypic effects, representing the variation’s exploitable portion. Therefore, selecting traits with high heritability can be more effective. However, heritability alone does not guarantee high genetic gain. It is essential to consider both heritability and genetic advance when selecting traits (Başçıftçi et al., 2013). Traits with high heritability and genetic advance indicate additive gene action’s influence (Gupta and Verma, 2000). In our study, we observed the high genetic advance of mean for traits such as spike length, peduncle length, tiller numbers, grain weight per spike, and grain yield, while plant height and thousand grain weight showed a moderate genetic advance of mean throughout the years. Traits with high heritability and low genetic advance suggest the effect of dominance and epistatic gene action (Gupta and Verma, 2000).

Cluster analysis using the BLUP estimates identified six distinct clusters with specific agronomic features, including days to heading, days to maturity, plant height, tiller number, grain number per spike, grain weight per spike, thousand grain weight, and grain yield. Cluster 1 comprised genotypes with shortest heading and maturity days but low yield. Cluster 2 included genotypes with the longest heading days and lowest thousand grain weight. Genotypes in Cluster 3 exhibited late maturity, taller plants, higher thousand grain weight, more tillers, and the second-highest grain yield. Cluster 4 grouped genotypes with the lowest tillers, yield, grain number per spike, and grain weight. Cluster 5 represented genotypes with the highest grain yield, grain number per spike, and tallest plants. Cluster 6 consisted of genotypes with the shortest plant height and average values for the other studied traits. Thus, genotypes with high tillers, higher grain number per spike and weight, and higher grain yield were grouped together, while genotypes with low tillers, lower grain number per spike and weight, and low grain yield but larger thousand grain weight were grouped separately. DWK26 and DWK38, belonging to Cluster 3 and 5, respectively, were released as Nepal’s first durum wheat varieties in 2017. The released varieties and their production technologies can be disseminated to meet the industrial demand and promote the country’s diversification of durum wheat products.

Regarding breeder seed production, based on the trend and availability of breeder seed, it is estimated that if the proper seed cycle is maintained, the C2 seed produced can be planted in 13.8% of the total wheat area in Nepal. However, the adoption of this variety at the farm level is currently very low, accounting for less than 0.1% of the total wheat area. The low adoption can be attributed to a lack of awareness about industrial wheat usage, unavailability of agro-based industries for Semolina, Macaroni, and Pasta production, and weak or nonexistent linkages between domestic agro-industries and Nepalese wheat farmers due to low production volume, which poses risks for regular raw material supply. While there is a possibility to increase durum wheat production, it is crucial to motivate and facilitate private seed companies, community-based organizations, and farmers to multiply seeds and utilize them for production, while maintaining the seed cycle and establishing linkages with agro-based industries to ensure sustainability through backward and forward linkages.

In conclusion, our comprehensive ten-year study of durum wheat genotypes introduced in Nepal has revealed substantial phenotypic and genotypic diversity among the accessions from CIMMYT lines. This genetic variation is pivotal for crop improvement efforts and the development of high-yielding, disease-resistant varieties suitable for diverse agricultural systems in Nepal. Through rigorous multiyear evaluations, we successfully identified stable and high-yielding durum wheat genotypes, leading to the release of Nepal’s first two durum wheat varieties, Khajura Durum 1 and Khajura Durum 2. These milestones mark a significant advancement in the country’s agricultural landscape, opening avenues for diversification of durum wheat products and meeting industrial demand. However, realizing the full potential of these varieties requires concerted efforts to raise awareness among farmers, establish robust linkages with agro-based industries, and promote seed multiplication. By addressing these challenges and harnessing the genetic potential of durum wheat, we can contribute to enhancing food security and sustainable agriculture in Nepal and beyond.

## Data Availability

The datasets presented in this study can be found in online repositories. The names of the repository/repositories and accession number(s) can be found in the article/**Supplementary Material**.
